# Gliosis and Neurodegenerative Diseases: The Role of PET and MR Imaging

**DOI:** 10.3389/fncel.2020.00075

**Published:** 2020-04-02

**Authors:** Carlo Cavaliere, Liberatore Tramontano, Dario Fiorenza, Vincenzo Alfano, Marco Aiello, Marco Salvatore

**Affiliations:** IRCCS SDN, Naples, Italy

**Keywords:** PET, MRI, gliosis, microglia, astrocytes, hybrid imaging, dual PET/MR agents, molecular imaging

## Abstract

Glial activation characterizes most neurodegenerative and psychiatric diseases, often anticipating clinical manifestations and macroscopical brain alterations. Although imaging techniques have improved diagnostic accuracy in many neurological conditions, often supporting diagnosis, prognosis prediction and treatment outcome, very few molecular imaging probes, specifically focused on microglial and astrocytic activation, have been translated to a clinical setting. In this context, hybrid positron emission tomography (PET)/magnetic resonance (MR) scanners represent the most advanced tool for molecular imaging, combining the functional specificity of PET radiotracers (e.g., targeting metabolism, hypoxia, and inflammation) to both high-resolution and multiparametric information derived by MR in a single imaging acquisition session. This simultaneity of findings achievable by PET/MR, if useful for reciprocal technical adjustments regarding temporal and spatial cross-modal alignment/synchronization, opens still debated issues about its clinical value in neurological patients, possibly incompliant and highly variable from a clinical point of view. While several preclinical and clinical studies have investigated the sensitivity of PET tracers to track microglial (mainly TSPO ligands) and astrocytic (mainly MAOB ligands) activation, less studies have focused on MR specificity to this topic (e.g., through the assessment of diffusion properties and T2 relaxometry), and only few exploiting the integration of simultaneous hybrid acquisition. This review aims at summarizing and critically review the current state about PET and MR imaging for glial targets, as well as the potential added value of hybrid scanners for characterizing microglial and astrocytic activation.

## Introduction

Selective cortical or subcortical atrophy, neuronal death and shrinkage characterize pathological feature across different neurodegenerative diseases and brain disorders (Chi et al., 2018).

Nevertheless, exclusively neuron-centric approaches to neuropathological phenomena have not returned new breakthroughs in the prevention and therapy of brain disorders (Verkhratsky et al., 2014). This gap is due to the complexity of neuronal networks that cannot only be explained by neuronal activity, addressing research efforts to different actors in the central nervous system (CNS): the glia (Papa et al., 2014).

Glial cells, mainly represented by microglia and astrocytes, are key components for development and maintenance of brain functions and circuitry. Microglial cells correspond to the brain-resident macrophage playing an essential role for synaptic pruning, CNS repair, and mainly, as cellular mediators of neuroinflammation that characterizes different brain disorders (Colonna and Butovsky, 2017). Astrocytes instead, initially deputed to support neuronal activity, have now gained a central role in brain function, for example being part of the concept of tripartite synapse and gliotransmission for their effects on neuronal communication and plasticity (Pérez-Alvarez and Araque, 2013).

Increasing evidences support the role of an altered glial function as an underlying dynamic feature in psychiatric and neurological disorders (Garden and Campbell, 2016).

As pharmaceutical efforts begin to focus on glial specific targets, a limiting step in knowledge consists of the unavailability of validated biomarkers to assess and monitor gliosis longitudinally, supporting clinical management and potentially identifying best responders to cells-specific drugs (Garden and Campbell, 2016).

Accordingly, the interest in the development of novel methods to investigate glial activation and, more in general, neuroinflammation, has surged over the past 15 years. Neuroimaging offers a wide panel of non- or minimally invasive techniques to characterize neuroinflammatory processes.

Among different imaging techniques, non-invasive brain functional measurements using positron emission tomography (PET) and detailed morpho-functional information provided by magnetic resonance imaging (MRI) appeared the more appealing for translational purposes, being both used in preclinical and clinical settings.

In this context, hybrid PET/MR scanner represent the most advanced tool for molecular imaging, combining the functional specificity of PET radiotracers (e.g., targeting metabolism, hypoxia, inflammation, and specific membrane receptors) to both high-resolution and multiparametric information derived by MR in a single imaging acquisition session.

Aims of this review are: to summarize preclinical and clinical studies employing both PET and MRI techniques to investigate glial contribute in brain disorders; to critically review the main evidences raised up by PET glial tracers in neurodegenerative disorders and their feasibility in a clinical context; to investigate the potential added value of hybrid PET/MRI for characterizing microglial and astrocytic activation in neurological and psychiatric diseases.

## Hybrid PET/MR Scanner

Compared to other more widespread and validated hybrid imaging techniques, like PET/computed tomography (CT), PET/MR scanner constitutes the first real effort to effectively integrate two modalities by simultaneous acquisition, going beyond the serial PET/CT, assuring naturally co-registered multimodal images (Monti et al., 2017). The integration and coregistration of multimodal information achieved by different imaging techniques is essential for a complete understanding of the brain processes, representing a crucial step both for visual qualitative assessment and for multiparametric quantitative analysis. The retrospective coregistration of complex diagnostic datasets serially acquired on different scanners is typically achieved via software, through transformation algorithms based on the anatomical information of the CT component of PET/CT. Despite of the cost effectiveness of this approach compared to hybrid solutions, retrospective coregistration could be particularly challenging and technically demanding in un-collaborative patients (Monti et al., 2017). This problem is intrinsically overcome by hybrid scanners that allow you to simultaneously acquire images that share the same coordinate system (Zaidi and Guerra, 2011).

This innovation has been possible after the development of dedicated hardware components for each modality, and not mutually influenced by the magnetic field or photon pathway (Aiello et al., 2018).

Nowadays, integrated PET/MRI represents the most advanced tool for molecular imaging, opening new insights for the characterization of neurological and psychiatric disorders, and possibly for a multifaceted patient management (Aiello et al., 2015, 2016; Cavaliere et al., 2018a). Indeed, this innovative clinical diagnostic scanner allows to combine the functional specificity of PET radiotracers (e.g., targeting metabolism, hypoxia, inflammation, specific ligands, or receptors) to both high-resolution and multiparametric information derived by MR in a single imaging acquisition session (Herzog et al., 2010; Aiello et al., 2019). This simultaneity of findings achievable by PET/MR, if useful for reciprocal technical adjustments regarding temporal and spatial cross-modal alignment/synchronization, opens still debated insights about its clinical value in neurological patients, possibly incompliant and highly variable from a clinical point of view (Zaidi and Guerra, 2011; Cavaliere et al., 2018b).

Nuclear medicine imaging techniques, like PET, offer the unique opportunity to investigate *in vivo* and in a mini- or not-invasively manner, a plethora of molecular mechanisms, depending on the selectivity of the radio-tracer used. This potentiality has pushed radio-chemists and drug industries to test and investigate new potentially innovative tracers able to specifically target pathophysiological pathways, such as neuroinflammation, glial cells or membrane receptors to disentangle neurodegenerative phenomena (Herzog et al., 2010). However, stand-alone PET imaging is still limited by low spatial resolution, that obliges it to be co-registered to an higher resolution imaging techniques, such as CT and MR. Compared to CT, MRI provides different quantitative information (e.g., water and metabolites diffusion, metabolites concentrations, regional perfusion, and activation), simply modifying sequences’ parameters, representing the gold standard for soft tissue and brain study and providing complementary information compared to PET imaging (Cavaliere et al., 2018b; Marchitelli et al., 2018). Moreover, the higher contrast resolution provided by MR can be more suitable for the segmentation of regions of reference useful to normalize PET signal in order to extract quantitative uptake parameters.

While, similarly to PET/CT scanners, findings derived by MRI and PET can be sequentially achieved in two different times, for highly dynamic systems like the brain this temporal gap could bias intermodality comparison. Indeed, sequential approach supposes that no significant modifications have occurred in the underlying state of the subject between two imaging sessions — a statement that could be ineffective both in physiological terms (e.g., changes in cognitive states or alertness) and in pathological conditions (e.g., psychiatric or neurological disorders) or as a result of medical interventions through psychological counseling, drugs or electrical/magnetic brain stimulations (Aiello et al., 2018; Cavaliere et al., 2018b). For these reasons, hybrid PET/MR scanners allow to overcome these limitations by simultaneously acquiring morphological, functional, molecular or metabolic information derived by both MRI and PET in a single shot.

## PET and MR Imaging of Microglial Activation

Neuroinflammation is a dynamic flogistic response which involve a complex multicellular cascade including the activation of both microglia and astrocytes, and the release of different neuroactive compounds.

Previous experimental studies have provided overwhelming evidence of the pivotal involvement of microglia-related molecular networks in the pathophysiology of many neurodegenerative and psychiatric diseases (Condello et al., 2018). However, the precise mechanisms by which microglia affect the disease’s progression and modify neuropathology remain poorly understood.

**FIGURE 1:**
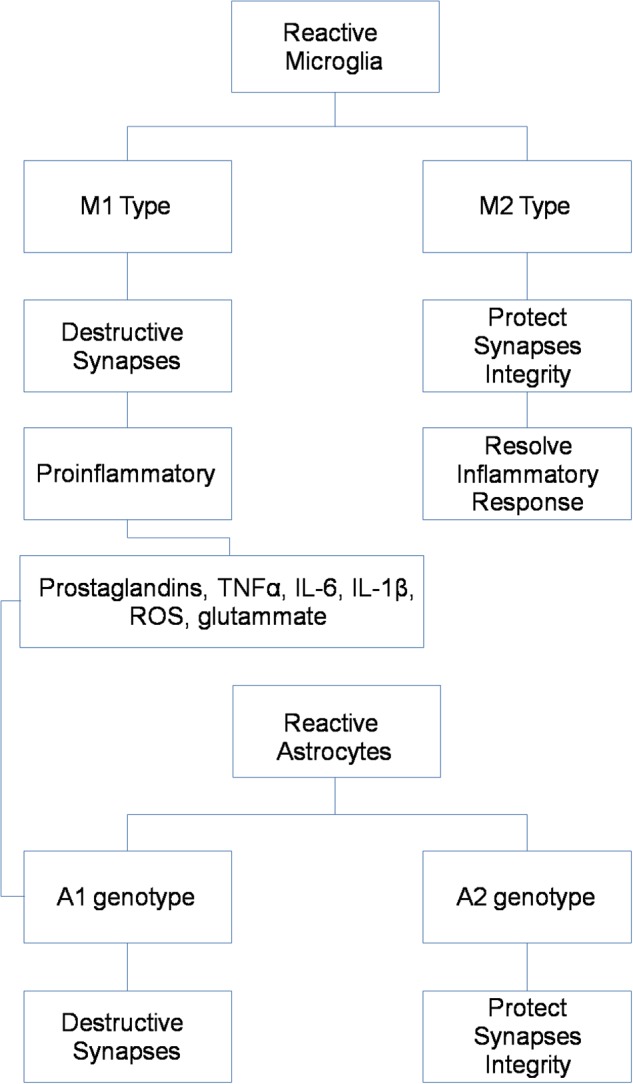
Microglial and astrocytic phenotypes as possible targets for *in vivo* PET/MR imaging.

Different activation phenotypes seem characterize chronic brain pathologies, ranging from a neuroprotective and anti-inflammatory M2 phenotype, mainly represented in an early stage, to the shift into the pro-inflammatory M1 phenotype, typical of later stages and contributing to neuronal dysfunction, injury, and disease progression (Figure 1) (Shen et al., 2018).

Therefore, the development of non-invasive molecular imaging methods able to dynamically characterize the spatio-temporal profile of neuroinflammatory biomarkers is widely attracting scientific and clinical interest, also for the application of immunomodulatory therapies both in clinical and preclinical settings (Narayanaswami et al., 2018).

**FIGURE 2:**
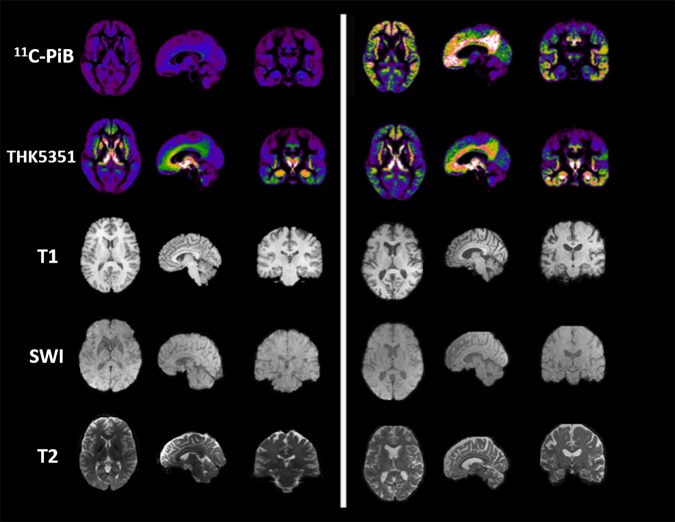
Imaging examples in PET and MR imaging. The figure depicts, from left to right, axial, sagittal and coronal projections of healthy **(Left)** and AD spectrum **(Right)** brain for different PET (Shigemoto et al., 2018) and MR contrasts. From top to bottom row: microglial uptake as revealed by 11C-PiB PET tracer; astrocytic uptake as revealed by 18F-THK5351 PET tracer; structural 3D T1-weighted MR used as anatomical reference for PET and to highlight cortical atrophy and ventricular enlargement; 3D Susceptibility-weighted imaging MR linked to iron deposition and microgliosis; T2-weighted MR scan used for T2 mapping and astrogliosis.

Positron emission tomography imaging of microglia has emerged over recent years, through the development of target-specific radiotracers and, among these, mainly the translocator protein-18 kDa (TSPO) (Table 1) (Figure 2).

**TABLE 1:** Main PET radiotracers used to investigate selective microglial and astrocytic activation.

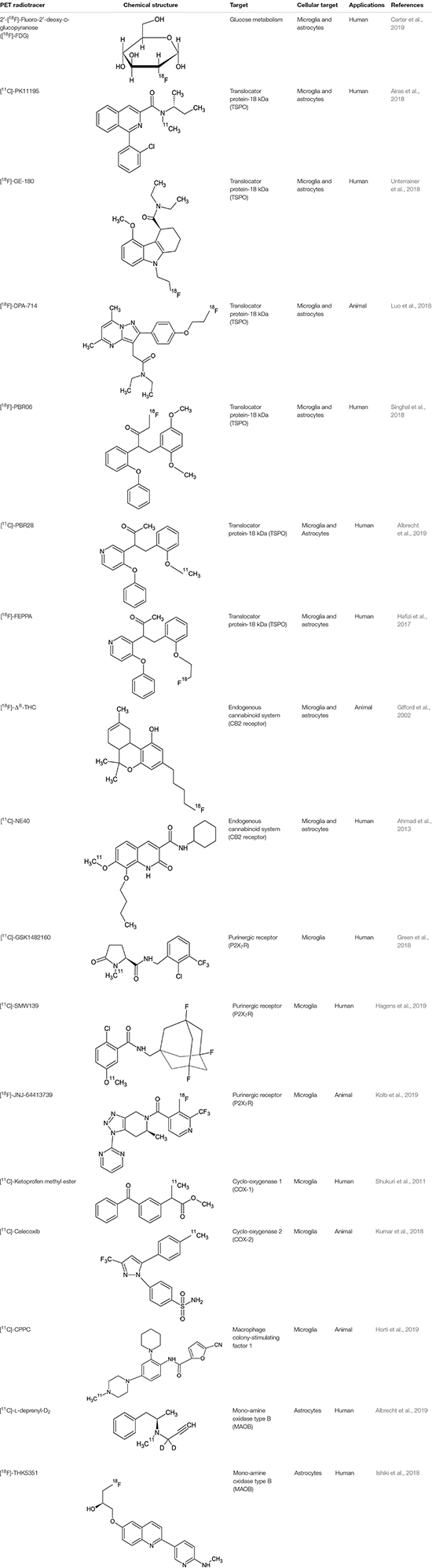

The first-generation radioligand [^11^C](R)-PK11195 has been the most widely studied in clinical studies and different neurodegenerative diseases, including multiple sclerosis (Airas et al., 2018), Creutzfeldt-Jakob disease (Iaccarino et al., 2018), and also in psychiatric disorders such as schizophrenia (Di Biase et al., 2017). Although the findings of an increased uptake of this tracer in these pathologies, limitations including low signal-to-noise ratio and high non-specific binding has addressed for the synthesis of second- and third-generation TSPO-specific radiopharmaceuticals, linked to [^11^C] or [^18^F] and including [^11^C]-PBR28, [^18^F]-DPA-714, [^18^F]-PBR06, [^18^F]-FEPPA, and [^18^F]-GE-180 (all used in a clinical context, except for [^18^F]-DPA-714) (Luo et al., 2018; Singhal et al., 2018; Best et al., 2019). Similarly, different studies have reported selective microglial uptake of these tracers in multiple sclerosis animal models and patients (Hagens et al., 2018; Herranz et al., 2019; Nack et al., 2019), amyotrophic lateral sclerosis (Zürcher et al., 2015; Datta et al., 2017), Alzheimer’s disease (Alam et al., 2017; Keller et al., 2018; Focke et al., 2019), and Lyme disease on humans (Coughlin et al., 2018), and stroke experimental models (Miyajima et al., 2018), with more discordant results for psychiatric patients, suffering from schizophrenia (Di Biase et al., 2017; Hafizi et al., 2017; Ottoy et al., 2018; Selvaraj et al., 2018) and major depression (Li et al., 2018), probably due to the different stage of disease. Interestingly, one study on fibromyalgia subjects attempts to demonstrate specificity of TSPO tracers for microglia, considering that an high expression of this molecule was also detected in activated astrocytes (Albrecht et al., 2019). The authors, using [^11^C]PBR28, which binds to the TSPO, and [^11^C]-DED thought to primarily reflect astrocytic (but not microglial) signal, demonstrated although in a small size sample, a selective cortical uptake of microglial tracer but not of the astrocytic one (Albrecht et al., 2019).

Nevertheless, the application of TSPO tracers is affected by significant inter-subjects variability, essentially due to a rs6971 polymorphism that affects TSPO binding mainly in first- and second-generation radioligands (Hafizi et al., 2017), and more importantly, they are not able to differentiate microglial phenotype, distinguishing between conservative and detrimental activation. Moreover, although TSPO is upregulated in activated glial cells, its function and role in the immunity response is still unclear. Finally, considering that major evidences emerged by TSPO uptake in multiple sclerosis patients, and the very low brain uptake in healthy subjects, several doubts have been raised for the blood–brain barrier permeability of these tracers in physiological conditions (Albert et al., 2019).

For this reason, other microglial targets have been identified for specific radiotracers in animal models, such as the cannabinoid receptor type 2 ([18F]-D8-THC) (Ni et al., 2019; Gifford et al., 2002), the P2X7 receptor ([^11^C]-GSK1482160) (Han et al., 2017), the cyclo-oxygenase 1 ([^11^C]-Ketoprofen methyl ester) (Shukuri et al., 2011), the cyclo-oxygenase 2 ([^11^C]-Celecoxib) (Kumar et al., 2018), and the macrophage colony-stimulating factor 1 ([^11^C]CPPC [5-cyano-N-(4-(4-[^11^C]methylpiperazin-1-yl)-2-(piperidin-1-yl) phenyl)furan-2-carboxamide]) (Horti et al., 2019).

Regarding the use of MRI for microgliosis identification, less studies have tried to directly characterize microglial activation in specific brain regions. Changes of iron deposition, quantified using quantitative susceptibility mapping in MR, have been correlated with activated microglia/macrophages at edges of some chronic demyelinated lesions in patients suffering from multiple sclerosis (Dal-Bianco et al., 2017; Gillen et al., 2018; Hametner et al., 2018). Advanced diffusion models, based on neurite orientation dispersion and density imaging (NODDI) in MR, have been proved to be sensitive to microglial density and to the cellular changes associated with microglial activation in a preclinical setting (Yi et al., 2019). Finally, in amyotrophic lateral sclerosis patients, a technique based on diffusion spectroscopy has been applied to identify the increase of the predominantly glial metabolites (unspecific for microglia and astrocytes) tCr (creatine + phosphocreatine) and tCho (choline-containing compounds) in the primary motor cortex (Reischauer et al., 2018).

Despite the proved potential of both PET and MRI for the microglial imaging, very few studies have integrated both the modalities in humans and, generally, the majority use MRI to simply colocalize TSPO-uptake with multiple sclerosis plaques identified by MRI (Colasanti et al., 2014; Kang et al., 2018; Unterrainer et al., 2018; Kaunzner et al., 2019). Up to now, only three studies performed a simultaneous PET/MRI to investigate microglial activation in neurodegenerative diseases. The first one, using a selective P2X7R radiotracers in patients affected by Parkinson’s disease, was not able to identify significant differences in PET uptake between patients and control subjects (van Weehaeghe et al., 2019). The second one, using a second-generation TSPO radiotracer in patients affected by multiple sclerosis, demonstrated microglial activation in both normal appearing cerebellum and segmented lesions (Barletta et al., 2019). The latter combines magnetic resonance spectroscopy of glial metabolites to TSPO-radiotracer uptake in patients affected by amyotrophic lateral sclerosis demonstrating a positive correlation between MR and PET biomarkers of neuroinflammation (Ratai et al., 2018) (Figure 2).

## PET and MR Imaging of Astrocytic Activation

Astrocytes are the most abundant cell type in the brain, and participate to complex neuronal-glial interactions to assure synaptic homeostasis and metabolic sustainment for neurons (Miller, 2018). As for microglial cells, different phenotypes seem characterize astrocytic activation in physiological and pathological conditions, ranging from a pro-inflammatory A1 phenotype to a protective and anti-inflammatory A2 phenotype (Figure 1) (Miller, 2018).

Previously considered only as a supporting neuronal cells, astrocytic contributions to different neurodegenerative and psychiatric diseases has been recently revised (Papa et al., 2014).

Compared to microglial visualization, PET radiotracers specific for astrocytic function have been only recently applied for clinical purpose, mainly binding the mono-amine oxidase type B (MAOB) enzyme, highly expressed by activated astrocytes (Table 1) (Figure 2) (Ishibashi et al., 2019). PET studies using the MAOB radiotracers 11C-DED or [18F]THK5351 in patients affected by Alzheimer’s disease have demonstrated an high correlation between brain tau deposition and astrocytic uptake (Harada et al., 2018) and between cortical glucose hypometabolism, detected by 18F-fluorodeoxyglucose [18F]-FDG PET, and longitudinal decline in astrocytic function detected by 11C-DED (Carter et al., 2019). These findings are in agreement with another clinical paper that demonstrate an increase of FDG uptake following selective activation of astrocytic glutamate transport (Zimmer et al., 2017). A specific regional increase of astrocytic function was also detected in two cases of progressive sopranuclear palsy studied with [18F]THK5351 PET (Ishiki et al., 2018).

Regarding the use of MRI to highlight astrocytic involvement in neurological and psychiatric diseases, few research groups have stressed MR potentiality to achieve this aim (Figure 2). Several preclinical studies proposed magnetic resonance spectroscopy to highlight lactate (Blanc et al., 2019) or acetate (Dehghani et al., 2017) peaks changes as surrogate markers specific for astrocytic metabolic homeostasis. However, magnetic field strength of clinically available scanner is not able to spectrally resolve these metabolites by surrounding peaks. In another clinical study (Alshelh et al., 2018), regional alterations in T2 relaxation times have been speculated as indicative of astroglial activation in patients suffered of neuropathic pain. More recently, the origin of signal used by functional MRI to investigate large-group of neuronal activations has been debated, considering preclinical evidences that evoked astrocytic Ca^2+^ waves can correlate with increased EEG power signal (Wang et al., 2018), astrocyte-evoked BOLD fluctuations (Takata et al., 2018), and transcranial direct current stimulation response (Monai and Hirase, 2018). These findings render furtherly more appealing the use of hybrid scanner that can acquire simultaneously PET, fMRI, and EEG signal (Mele et al., 2019).

Anyway, evidences derived by MRI on astrocytic activation are still confined to few groups, and often on limited samples. Few studies have serially used both PET and MRI with this purpose, and employing MR findings to colocalize PET uptake. In a longitudinal study on patients affected by corticobasal syndrome, [18F]THK5351 binding have been demonstrated to detect dynamical astrogliosis in specific cortical regions (Ezura et al., 2019). In a case of multiple sclerosis, instead, the authors demonstrated the coregistration of [18F]THK5351 uptake with demyelinating plaques (Ishibashi et al., 2019). Another study compared the spatial uptake patterns of [18F]THK5351 and fMRI network alterations in patients with early AD and healthy controls, showing a similar pattern for the precuneus, a region crucial for Alzheimer progression (Yokoi et al., 2018).

Finally, in patients suffering of semantic variant primary progressive aphasia, several authors demonstrated that the MAOB tracers can be more sensitive to detective neurodegenerative alterations compared to MRI (Kobayashi et al., 2018).

## Glial PET Radiotracers: a Critical Point of View

Despite the remarkable development of radiotracers for numerous molecular targets implicated in the process of neuroinflammation, only a few have been used on patients successfully.

### TSPO PET Tracers

The main problems of TSPO tracers are that they cannot distinguish pro- and anti-inflammatory responses, they have a low signal-to-noise ratio and high non-specific binding, low dynamic response variation during neurodegenerative pathologies (Figure 2). Furthermore the main problem in the clinical use of these PET tracers is given the presence of polymorphism (SNP) in TSPO gene. These problems have been reduced and/or eliminated with second and third generation TSPO PET tracers, however, there is still no possible differentiation between the M1 (neurotoxic) and M2 (neuroprotective) genotypes. Moreover, even if the 3D pentameric structure of the TSPO has been revealed, the role of this receptor does not yet appear to be clear, since the influence of receptor upregulation as an immune reaction is not clear. Currently [18F] -GE180 is used as a third-generation tracer in clinical trials. Although [18F] -GE180 is able to pass in CNS only in case of blood–brain barrier breakdown, with low BBB permeability in human healthy brain, this radiotracer is a very important neuroinflammation imaging agent in various CNS diseases (Albert et al., 2019).

### Cannabinoid Receptor Type 2 PET Tracers

Due to its high brain density, this receptor has been subjected to *in vivo* imaging using PET techniques using radioligands, such as Δ^8^-Tetrahydrocannabinol (Δ^8^-THC) labeled with fluorine-18. However, the PET images obtained after injection of [18F]-Δ^8^-THC in primates do not show a particular specific region of cerebral localization, so much so that the radiotracer appears to be widely diffused in all regions of the brain (Gifford et al., 2002). Some of the synthetic derivatives of classic cannabinoids, which show nano affinity for CB2 receptors, may prove to be successful candidates for *in vivo* imaging of these receptors, including pyrazole derivatives and aminoalkylindole derivatives. Since the level of expression of CB2 receptors in the healthy brain is low and there is an increase of these receptors in pathological conditions, much research has focused on these receptors. To date only the radiotracer [^11^C] -NE40 has been used in humans for biodistribution studies on healthy patients, to verify the uptake and washout (Ahmad et al., 2013). CB2 ligands are still in the preclinical evaluation phase, however, they can represent a valid alternative in the evaluation of microglial activation (Yamagishi et al., 2019).

### Cyclooxygenase-2 PET Tracers

Despite the potential given by the study of the increase in cyclooxygenases (COX1 and COX2) in inflammation, actually only very few radiotracers have been synthesized and studied. For neuroinflammation studies it is mandatory to use selective radioligands for the respective isoforms COX1 and COX2. The advantage in the use of [^11^C]-celecoxib is certainly represented by the ability to pass the blood–brain barrier, even when it is not damaged (Kumar et al., 2018).

### P2X_7_ Receptor PET Tracers

P2X7 receptor is an adenosine triphosphate ATP-gated purinoreceptor that is widely expressed in microglia and astrocytes. The activation of P2X7 receptor leads to the release of the proinflammatory mediators such as cytokine IL-1β in the brain. In fact the increase in the expression of this receptor leads to the activation of microglia, more than its over-expression is a consequence of the activation of microglia. Over the last years, research has focused on the synthesis and study of ligands of this receptor. The most promising are certainly: [^11^C]-GSK1482160, [^11^C]-SMW139, and [^18^F]-JNJ-64413739. The first [^11^C]-GSK1482160 has been studied in healthy volunteer patients for the estimation of radiopharmaceutical radiation dosimetry and biodistribution (Green et al., 2018). The first PET/MRI study, even if preliminary, on patients affected by multiple sclerosis was conducted in 2019 with the administration of [^11^C]-SMW139 (Hagens et al., 2019). The Janssen compound [^18^F]-JNJ-64413739 is labeled with fluorine-18, which certainly represents an advantage from a clinical point of view considering the longer radioisotope half-life (Kolb et al., 2019). [^18^F]-JNJ-64413739 is still under evaluation in preclinical study phase and data on patients are not yet available, however, the first studies on animals show its potential as a PET tracer for neuroinflammation.

### Macrophage Colony-Stimulating Factor 1 Receptor (CSF1R) PET Tracer

CSF1R is a surface receptor (tyrosine kinase family receptors) mainly expressed by microglia. Recently a specific radioligand of this receptor was synthesized and studied, [^11^C]-CPPC (Horti et al., 2019). Even if this PET ligand is still in a preclinical phase of animal experiments, this new class of radioligands could be very promising for the study of microglial PET/MRI activation.

### Monoamine Oxidase-B PET Tracers

During neuroinflammation, astrocytic increased MAO-B (amine oxidases) activity which leads to an oxidative stress due to the formation of hydrogen peroxides (Gulyás et al., 2011). Among PET ligands MAO-B studied and synthesized over the years, two radiotracers were successfully used in patients, one of which containing carbon-11([^11^C]-L-deprenyl-D2) (Arakawa et al., 2017), the other containing fluorine-18 ([^18^F]-THK5351) (Ishibashi et al., 2019). The last one was used in a patient with relapsing-remitting multiple sclerosis (MS), it was seen that [^18^F]-THK5351 accumulation corresponded to sites identified in MRI where there were MS plaques, in lesions undergoing astrogliosis.

## Bimodal Probes as Future Perspectives

Although PET/MRI has been often addressed as the new frontier of molecular imaging (Chen and Chen, 2010), very few studies have employed this technique in glial imaging, and often limiting MR to a structural reference for PET signal. One possible innovation in this field could be represented by the development of an efficient and reliable PET/MR dual imaging probe, that if challenging for chemists and neuroscientists (Bouziotis et al., 2013; Vecchione et al., 2017), can help to identify killer applications for PET/MRI.

A general issue for new single- or multi-modal imaging probes that target the brain and that have to be taken into account is the permeability of the blood–brain barrier, a complex multicellular structure that protect CNS by external neurotoxic substances and which functions are assured also by astrocytic endfeet (Poupot et al., 2018). A possible solution is represented by the use of physiological transfer existing across the barrier, like the use of specific carriers or the transcytosis phenomenon (Pulgar, 2019).

Among novel imaging tools, different nanoparticles (usually smaller than 100 nm) have been extensively used in preclinical settings as MRI contrast agent combined with a PET tracer, mainly for oncological and more recently cardiovascular imaging (Aime et al., 2002; Uppal et al., 2011; Lewis et al., 2015; Kirschbaum et al., 2016; Vecchione et al., 2017; Grimaldi et al., 2019). Recently, a potential multimodal PET/MRI probe has been described to target microglia and neuroinflammation in a mouse model (Tang et al., 2018). The proposed nanoparticle selectively binds to the scavenger receptor class A (SR-A) expressed on activated microglia and is iron-oxide coated and so detectable by T2^∗^ -weighted MRI.

## Conclusion

In this review main PET and MR imaging biomarkers for microglial and astrocytic activation have been summarized. Furthermore, these studies also demonstrate potential benefits for the integration of findings achievable by simultaneous PET/MRI scanners, although still few employed in the literature. While the technological challenges seem to be overcome with new powerful scanners able to *in vivo* characterize molecular processes, more efforts to identify selective glial targets and efficient multimodal imaging probes, potentially useful for tailored treatments targeting glial activation, are needed.

## Author Contributions

CC designed and wrote the manuscript. LT, DF, and VA wrote the manuscript and prepared figures/tables. MA and MS revised and approved the text.

## Conflict of Interest

The authors declare that the research was conducted in the absence of any commercial or financial relationships that could be construed as a potential conflict of interest.
